# Transcervical approach to distal extracranial internal carotid aneurysm

**DOI:** 10.1016/j.jvscit.2024.101463

**Published:** 2024-02-27

**Authors:** Joseph AbouAyash, Benjamin Greif, Gregory Salzler, Evan Ryer, Robert Garvin

**Affiliations:** a Geisinger Commonwealth School of Medicine, Scranton, PA; b Department of Vascular and Endovascular Surgery, Geisinger Medical Center, Danville, PA

**Keywords:** Distal extracranial internal carotid aneurysm, Internal carotid aneurysm, Open repair, Transcervical approach

## Abstract

We report the case of a 77-year-old woman presenting with an asymptomatic internal carotid artery (ICA) aneurysm arising at the skull base. The distal right extracranial ICA aneurysm presented as a challenging case due to difficulty in obtaining adequate surgical exposure and preserving the facial nerves present near the ICA aneurysm. Transcervical open repair with a team of vascular and otolaryngology surgeons was completed successfully. In this report, we detail the operative steps needed to complete this exposure and our perioperative management.

Internal carotid artery (ICA) aneurysms are rare. It is currently estimated that 0.1% to 2% of all carotid artery procedures are performed due to aneurysmal disease.^1^ The anatomic constrictions for distal surgical exposure require seldom used techniques, including mandibular subluxation and lateral mandibulotomy.^2^ Additionally, the risk of cranial nerve injury (CNI) is high.^3^ Previously reported open repair approaches have yielded satisfactory long-term outcomes. Yet, due to their high degree of invasiveness, such as in the lateral skull base approach, complications such as facial nerve palsy and conductive hearing loss are not uncommon.^4^, ^5^, ^6^

We present the case of a 77-year-old woman with a right-sided extracranial carotid aneurysm (ECCA) located at the base of the skull. Open resection of the aneurysm with primary anastomosis was performed. In this report, we describe the operative technique used to approach the ICA at the base of the skull. The patient provided written informed consent for the report of her case details and imaging studies.

## Case report

**Fig 1:**
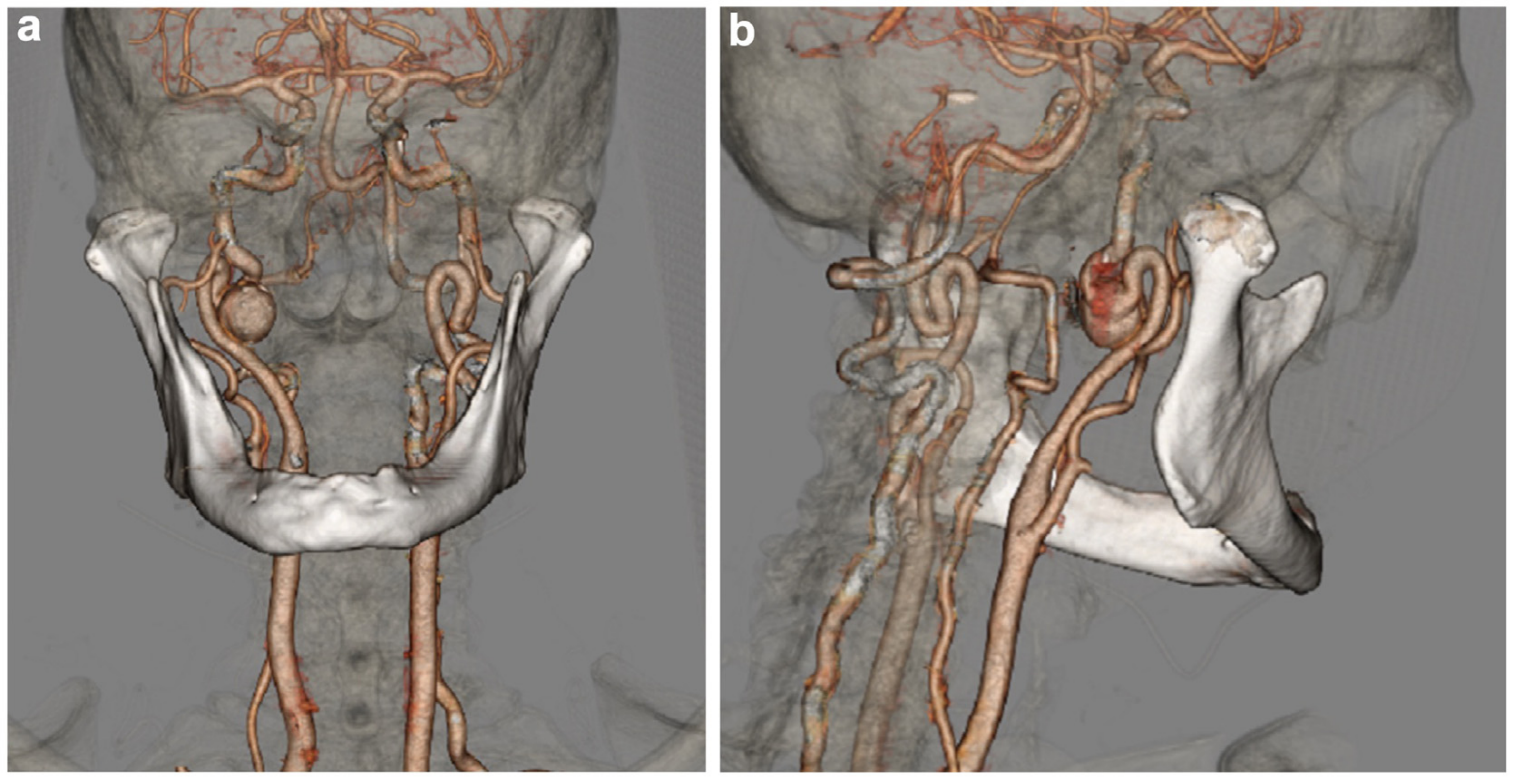
Three-dimensional computed tomography images of the aneurysm. **A,** Anterior view. **B,** Posterolateral right view.

A 77-year-old woman presented with an incidentally discovered asymptomatic distal cervical right ICA aneurysm. On computed tomography angiography (CTA), the aneurysm had no luminal thrombus, measured 17 mm in diameter, and had a normal caliber artery measuring 5 mm in diameter proximally and distally to the aneurysm (Fig 1). The artery proximal to the aneurysm was severely tortuous with two sharp, hairpin 180° turns in the artery, making the prospect of endovascular repair unrealistic. Thus, the decision was made to begin with open repair in combination with a team of otolaryngology colleagues.

**Fig 2:**
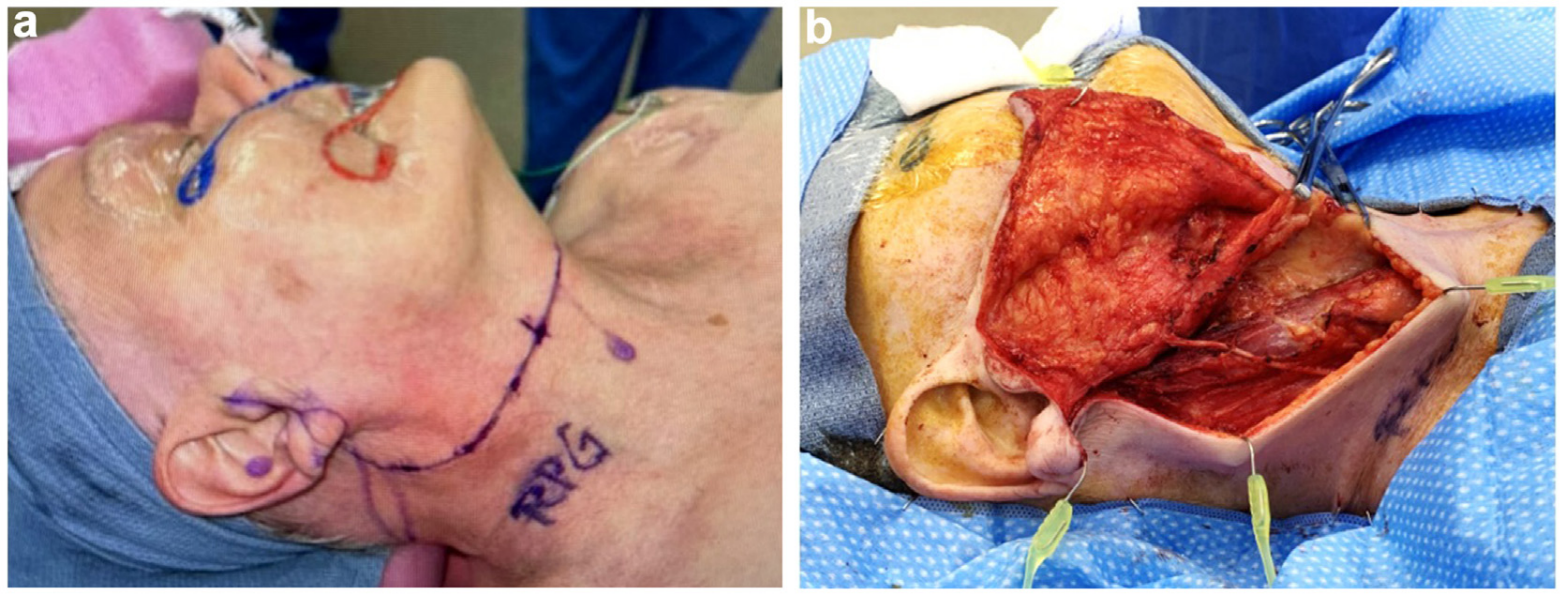
**A,** Facial markings before surgery. **B,** Facial dissection.

Under general endotracheal anesthesia, a modified Blair incision was made and carried down to the level of the platysma (Fig 2). Superior and inferior subplatysmal flaps were raised. Once the anterior border of the sternocleidomastoid muscle was skeletonized, further dissection in level II of the neck was performed, tracing the posterior belly of the digastric muscle back to the mastoid process. Using the tragal pointer and the digastric muscle as a landmark, the main trunk of the facial nerve was identified medially and traced forward to the pes anserinus. Upper and lower branches were identified and preserved. The parotid artery was then dissected off the nerve and explanted.

The spinal accessory nerve was identified and traced along its course to the posterior belly of the digastric muscle, which was divided to further observe the hypoglossal nerve. We then worked more cephalad to expose the glossopharyngeal nerve, which was followed toward the base of the skull. The internal jugular vein was skeletonized, and the facial vein was divided. The level IIa lymph packet was dissected from laterally to medially until the vessels were exposed.

**Fig 3:**
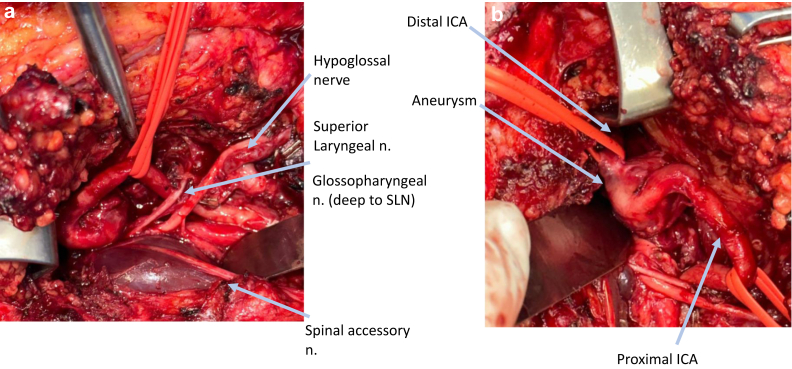
Close-up images during open repair. **A,** A review of the neurovascular structure surrounding the aneurysm. **B,** Close-up of the high right internal carotid artery (ICA) aneurysm.

After partial release of the sternocleidomastoid muscle from its mastoid attachment, the posterior belly of the digastric muscle was divided, along with the distal external carotid artery. The ICA was identified near the bifurcation and traced distally toward the aneurysm. The styloid musculature and stylomandibular ligament were divided, followed by resection of the styloid bone and distraction of the mandible with a bone hook. As dissection proceeded along the ICA, cranial nerves IX, X (including the superior laryngeal nerve), XI, and XII were protected and preserved (Fig 3, *A*). The aneurysm was dissected free from the surrounding soft tissue attachments, bringing the distal ICA into view (Fig 3, *B*).

**Fig 4:**
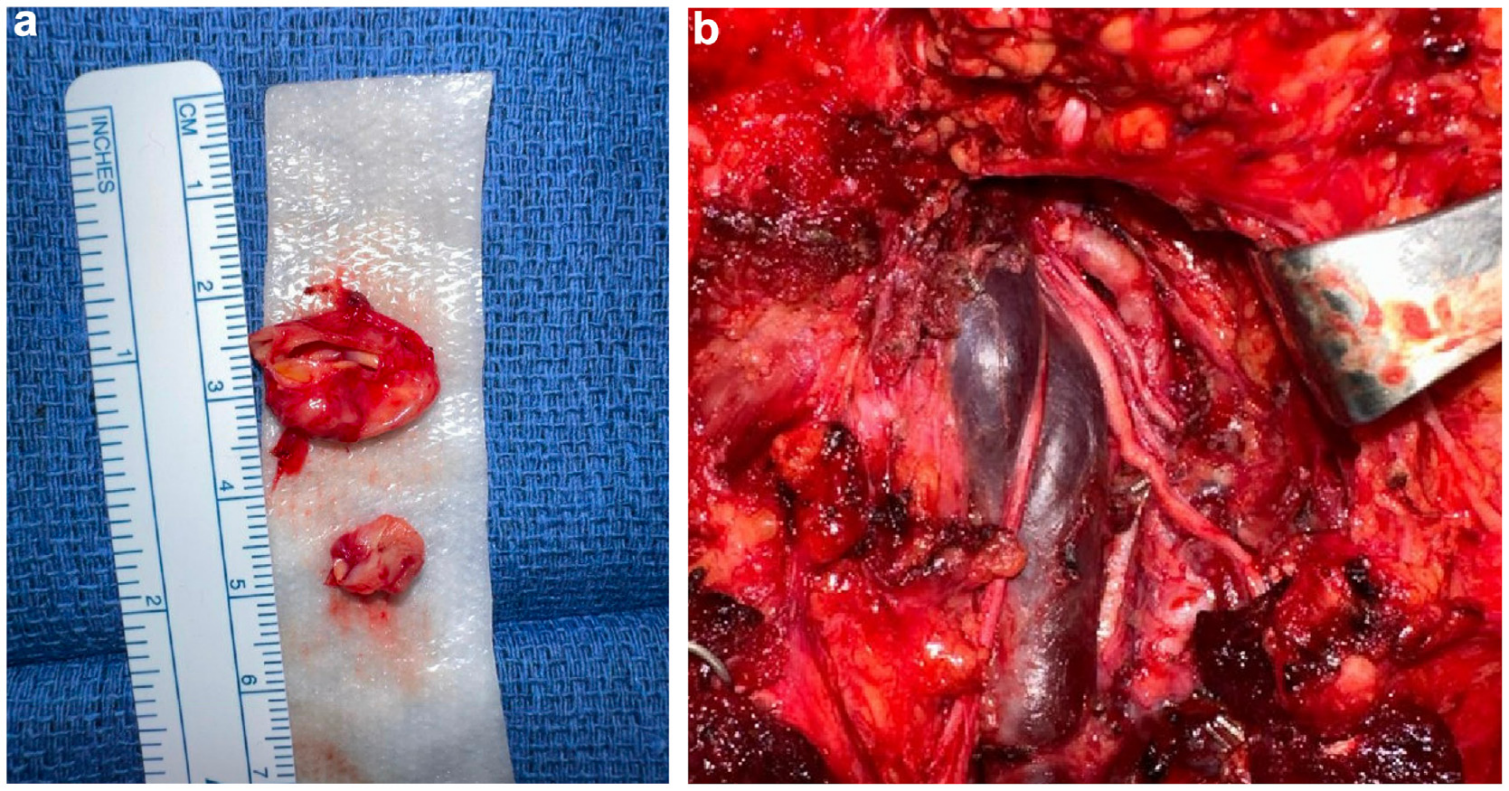
**A,** Off field view of resected aneurysm with ruler. **B,** Reconstructed artery anastomosis in a primary end-to-end anastomosis.

With this exposure, the space was adequate for proximal and distal clamping, which would leave only a short cuff of artery distally before entering the skull base. Therefore, the procedure was done without a shunt (so as not to potentially lose control of the artery distally), and the systolic blood pressure was maintained at >150 mm Hg during clamping. Intravenous heparin was administered, and the artery was clamped proximally and distally to the aneurysm. The aneurysm was resected, leaving a short, partially spatulated distal cuff. The tortuous proximal end of the artery was mobilized and trimmed to length, then slightly spatulated, and sewn in end-to-end fashion to the distal stump using running 7-0 Prolene suture (Fig 4).

The initial postoperative course was uncomplicated. The patient had a partial right-sided facial nerve palsy, as expected, with some difficulty in swallowing for the first 48 hours. She was cleared by speech pathology for a regular diet on postoperative day 3 before being discharged home later that day.

The patient returned to the hospital on postoperative day 5 after a brief episode of left upper extremity weakness that resolved before her arrival at the hospital, which we considered a transient ischemic attack. Cross-sectional imaging with CTA showed nonocclusive thrombus at the distal right ICA anastomotic site, likely responsible for her transient ischemic attack. The decision was made to manage the thrombus nonoperatively. Oral clopidogrel 75 mg once daily was administered, along with full anticoagulation using unfractionated heparin. The patient experienced no further symptoms and was discharged home 3 days after admission with an oral regimen of aspirin 81 mg once daily, clopidogrel 75 mg once daily, and apixaban 5 mg twice daily. Repeat CTA on the day of discharge demonstrated partial resolution of the thrombus. Another CTA at 1 month showed complete resolution of the thrombus. The apixaban was discontinued at the 6-month follow-up visit, at which time, there was complete resolution of the thrombus. The patient is currently maintained with dual antiplatelet therapy with 81 mg of aspirin and 75 mg of clopidogrel once daily.

## Discussion

The most common cause of an ECCA is atherosclerosis. However, other etiologies include trauma, fibromuscular dysplasia, congenital defects, prior surgery, infection, and radiation.^7^^,^^8^ There is a current debate on whether an open surgical or endovascular approach is most appropriate for repair and regarding the size criteria indicating the necessity of repair. Regardless of the approach, repair is generally recommended when the aneurysm is ≥2 cm in size or ≥1.5 times the diameter of the normal adjacent artery due to the increased risk of neurologic symptoms or rupture.^9^, ^10^, ^11^

Historically, open repair was the main choice for treatment of ECCAs. In contrast, today endovascular repair seems to be the favored approach if the anatomy is suitable. A recent systematic review by Hoffman et al^12^ reported that of 750 ECCA open repairs, there were 68 (9%) CNIs, 27 (4%) perioperative strokes, and 18 (2%) perioperative deaths. Of the 85 ECCAs treated with endovascular repair, no associated postoperative CNIs, 30-day strokes, or 30-day mortalities occurred.^12^ Li et al^13^ suggest that the indications for endovascular repair would be a distal location and necks with a hostile anatomy. In our patient, the degree of tortuosity excluded stenting as a viable option; thus, open repair was undertaken.

## Conclusions

We report a case of a successful open repair of a distal right ICA aneurysm at the base of the skull. The key steps of the operative approach and relevant anatomy are outlined. With the scarcity of distal carotid exposure in modern vascular surgery, we hope our operative description serves as a review of an uncommon exposure for future cases.

## Disclosures

None.
